# A Novel Out-of-Control Action Plan (OCAP) for Optimizing Efficiency and Quality in the Wafer Probing Process for Semiconductor Manufacturing

**DOI:** 10.3390/s24165116

**Published:** 2024-08-07

**Authors:** Woonyoung Yeo, Yung-Chia Chang, Liang-Ching Chen, Kuei-Hu Chang

**Affiliations:** 1 Department of Industrial Engineering and Management, National Yang Ming Chiao Tung University, Hsinchu 300, Taiwan; 2 Department of Foreign Languages, R.O.C. Military Academy, Kaohsiung 830, Taiwan; 3 Department of Management Sciences, R.O.C. Military Academy, Kaohsiung 830, Taiwan

**Keywords:** out-of-control action plan (OCAP), wafer probing process, semiconductor manufacturing, probe test yield, overall equipment effectiveness (OEE)

## Abstract

The out-of-control action plan (OCAP) is crucial in the wafer probing process of semiconductor manufacturing as it systematically addresses and corrects deviations, ensuring the high quality and reliability of semiconductor devices. However, the traditional OCAP involves many redundant and complicated processes after failures occur on production lines, which can delay production and escalate costs. To overcome the traditional OCAP’s limitations, this paper proposes a novel OCAP aimed at enhancing the wafer probing process in semiconductor manufacturing. The proposed OCAP integrates proactive measures such as preventive maintenance and advanced monitoring technologies, which are tested and verified through a comprehensive experimental setup. Implementing the novel OCAP in a case company’s production line reduced machine downtime by over 24 h per week and increased wafer production by about 23 wafers per week. Additionally, probe test yield improved by an average of 1.1%, demonstrating the effectiveness of the proposed method. This paper not only explores the implementation of the novel OCAP but also compares it with the traditional OCAP, highlighting significant improvements in efficiency and production output. The results underscore the potential of advanced OCAP to enhance manufacturing processes by reducing dependency on human judgment, thus lowering the likelihood of errors and improving overall equipment effectiveness (OEE).

## 1. Introduction

The development of semiconductor manufacturing has been pivotal in advancing artificial intelligence (AI). This process involves creating integrated circuits (microchips) used in electronic devices through photolithographic and chemical techniques [1,2,3]. It has significantly contributed to technology by enabling the miniaturization and enhancement of electronic components, essential in consumer electronics, automotive systems, telecommunications, and healthcare devices [4]. The semiconductor manufacturing process is generally divided into two main stages: frontend and backend manufacturing [5,6,7]. Frontend manufacturing includes wafer fabrication and wafer probing, where electronic circuits are built layer by layer on the wafer, with excess material removed [8]. Backend manufacturing involves dicing the wafer into individual chips and assembling them into various packages [9]. This stage includes packaging, which connects electrical signals within the chips, and testing, which ensures the chips’ electrical functionality. Wafer probing is a crucial quality control step performed after the wafer bumping process and before chip dicing. During this step, probe pins make contact with the chip pads to test the circuit’s performance and characteristics [10,11,12].

Wafer probing is a critical diagnostic step in semiconductor manufacturing where a specialized machine tests each chip on a silicon wafer by making physical contact with the chip pads using fine probe pins [13]. This process verifies the electrical functionality and performance of the circuits before the wafers are cut into individual chips. Identifying and discarding defective chips at this stage ensures the reliability and quality of semiconductor devices, reducing costs and improving yield [14,15]. Maintaining stable control during the wafer probing process directly influences the test yield [16]. Process engineers continually work to enhance yield and optimize conditions to ensure consistency [17]. Low yields during wafer probing can substantially affect manufacturing efficiency. Yeo et al. [18] identified significant factors impacting yield, including the overdrive of pogo pins, the frequency of online clean touchdowns, and the dimensions of the clean sheet [19]. They analyzed troubleshooting data to explore the relationship between yield and various probing settings, ultimately presenting optimal conditions to improve yield and overall equipment effectiveness (OEE). Sinhabahu et al. [20] highlighted the importance of the thorough cleaning of probing needles (pogo pins) to achieve high precision in wafer testing. However, current practices lack the preemptive detection of embedded debris, contributing to yield loss and inefficiencies. Over-cleaning also diminishes needle lifespan. They proposed a framework using real-time imaging and die contact resistance, incorporating image processing and supervised machine learning, to detect foreign material buildup and monitor needle degradation, enhancing cleaning effectiveness and precision. Sinhabahu et al. [21] emphasized the critical need for maintaining clean pogo pins for circuit probing, as continuous testing leads to contamination, affecting measurement quality. They identified three main challenges: a reliance on experience and reactive measures, a lack of predictive debris detection, and the detrimental effects of over-cleaning. Their proposed framework uses supervised machine learning for real-time analysis of die contact resistance to detect foreign material buildup and monitor needle degradation, aiming to improve yield and extend probe life. In this context, the out-of-control action plan (OCAP) is vital for managing deviations in the wafer probing process. When anomalies are detected, OCAPs guide operators through troubleshooting and corrective steps to restore process stability. This systematic approach ensures high accuracy and efficiency in wafer probing, safeguarding the manufacturing workflow from costly disruptions and quality issues. By applying OCAPs, semiconductor facilities can swiftly and effectively address operational outliers, optimizing both the reliability of the wafer probing results and overall production yield. OCAPs are predefined procedures initiated when a manufacturing process deviates from its control limits. They help operators quickly identify the cause of the deviations and take corrective actions to bring the process back under control. The main goals of an OCAP are to mitigate process variations, prevent nonconforming products, and ensure efficient and effective operations. OCAPs are crucial in maintaining high quality and yield in complex manufacturing environments, minimizing downtime, and reducing costs associated with defects and rework.

However, traditional OCAPs are primarily reactive, addressing issues after they occur, which can lead to delays, quality problems, and financial losses. These processes are complex and resource-intensive, requiring well-trained personnel and robust systems. They rely heavily on human judgment, increasing the risk of errors and biases, and often focus on immediate problems without addressing broader systemic issues. To address these issues, this paper proposes a novel OCAP with proactive measures, including preventive maintenance, continuous improvement initiatives, and advanced monitoring technologies. Investing in training, communication channels, and data analytics capabilities can optimize the effectiveness of the proposed novel OCAP in managing out-of-control situations, enhancing production line efficiency and capacity.

## 2. Related Works

### 2.1. Wafer Probing Process

Wafer probing, or wafer testing, stands as a pivotal phase in the semiconductor manufacturing journey, merging science with technological prowess to guarantee the quality and functionality of semiconductor devices [22]. The process can be described as:

Step 1. Application of electrical signals.

**Figure 1:**
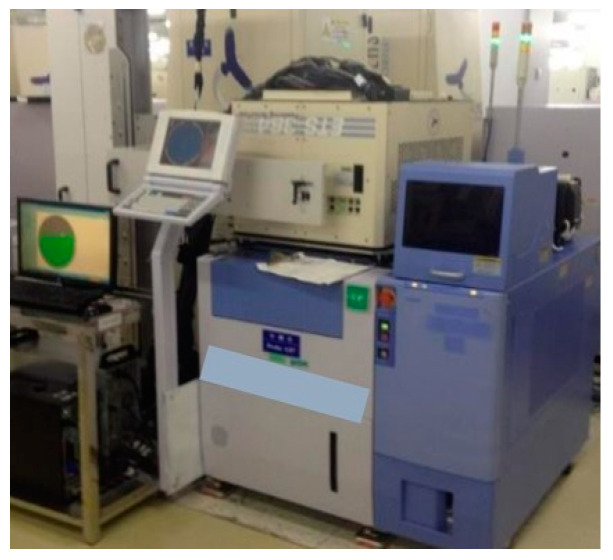
Wafer probe station.

**Figure 2:**
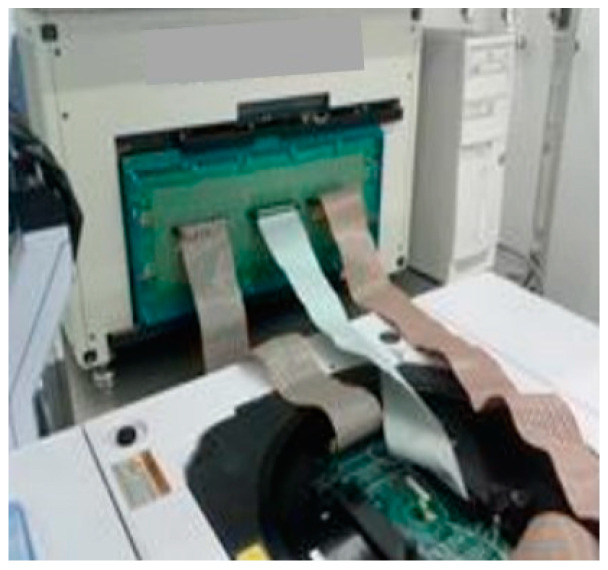
Wafer probers.

Electrical signals are applied to a semiconductor wafer’s circuitry using sophisticated equipment known as wafer probers or probe stations (see Figure 1 and Figure 2) [23].

Step 2. Use of probes to evaluate integrated circuits (ICs).

**Figure 3:**
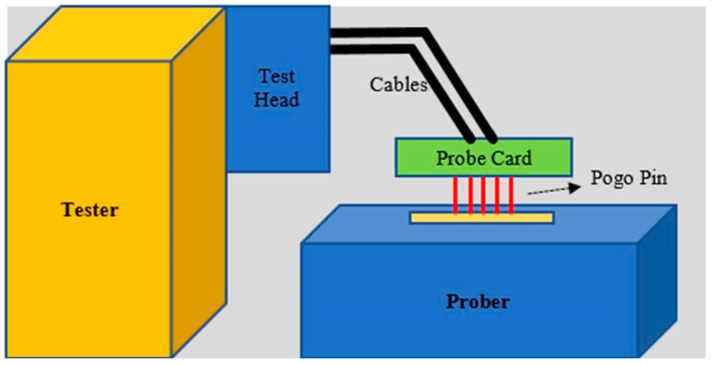
Scheme of wafer probing process.

These devices are equipped with probes designed to touch the wafer’s surface, enabling the evaluation of the integrated circuits’ (ICs) performance, integrity, and overall functionality (see Figure 3) [24].

Step 3. Timing of the process.

Wafer probing is typically undertaken after the semiconductor devices have been fabricated on a wafer but before the wafer is segmented into individual chips [24].

Step 4. Objective of wafer probing.

The core objective is to identify any defects, faults, or malfunctions in the ICs [25].

Step 5. Ensuring functional chips.

Only fully functional chips are allowed to advance to subsequent stages of assembly and packaging [22].

Step 6. Insights into electrical characteristics.

Wafer probing provides valuable insights into the electrical characteristics of the ICs, such as voltage, current, resistance, capacitance, and frequency response [24].

Step 7. Guidance for further steps.

The insights gained from this testing phase guide further steps in the semiconductor manufacturing process, including binning, packaging, and the final rounds of testing [25].

On the frontier of research, wafer probing is a hotbed of innovation aimed at pushing the boundaries of efficiency, accuracy, and reliability in testing to keep pace with the evolving landscape of semiconductor technologies [26]. Researchers are delving into new probing techniques, such as multi-site probing and the development of advanced probe materials, to boost throughput and minimize potential damage to the wafer’s intricate circuitry [21]. The exploration of methods to test more complex semiconductor designs, including three-dimensional integrated circuits (3D ICs) and nascent fields like silicon photonics, is also underway [24,27]. Addressing challenges such as contact resistance, signal integrity, and the seamless integration of wafer probing with other testing and inspection methods is of paramount importance [21,25,26]. Through these efforts, wafer probing not only ensures the performance and quality of semiconductor devices but also drives technological advancements across various sectors, including electronics, telecommunications, automotive, microdisplay technology, and consumer electronics [22,23,28]. This underscores its critical importance in the modern technological era.

### 2.2. Current Wafer Probing Process along with the Traditional OCAP

**Figure 4:**
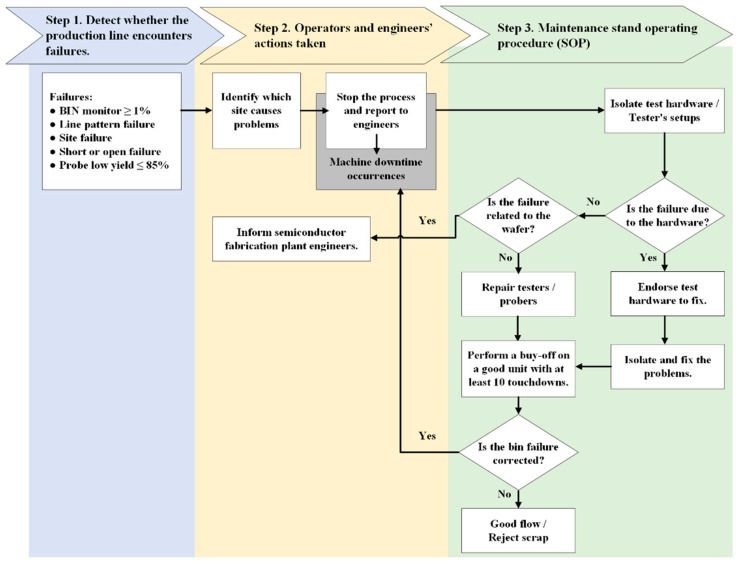
Flowchart of the traditional OCAP.

Taking a semiconductor manufacturing plant in Taiwan as an example, the traditional OCAP is adopted to assist operators or engineers in swiftly pinpointing the cause of deviations and implementing corrective measures to restore process control. The traditional OCAP can be divided into three major steps (see Figure 4), which are explained as follows:

Step 1. Detecting whether the production line encounters failures.

The production line faces a failure situation when reaching any of the following conditions:

- Bin monitor ≥ 1%.
- Line pattern failure.
- Site failure.
- Short or open failure.
- Low probe yield, ≤85%.

**Figure 5:**
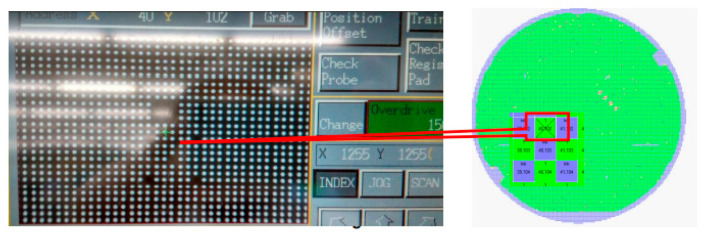
An example of wafer probing test failure (line pattern).

A bin refers to a storage area in which data is recorded during a wafer probing test. Generally, when a wafer is removed from the normal wafer probing process and placed into a “bin” for a specific reason, it indicates that the wafer is an abnormal product. Reasons for binning a wafer may include, for example, line pattern failures, site issues, or open/short situations. A line pattern refers to a distinct arrangement of test signals applied to the chip during its examination. These line patterns target particular functionalities of the chip for testing. A failure in meeting a line pattern criterion during wafer probe testing could suggest a defect in the chip or a problem with the OCAP process flow. Figure 5 shows an example of the wafer probing test when line pattern failure is found.

**Figure 6:**
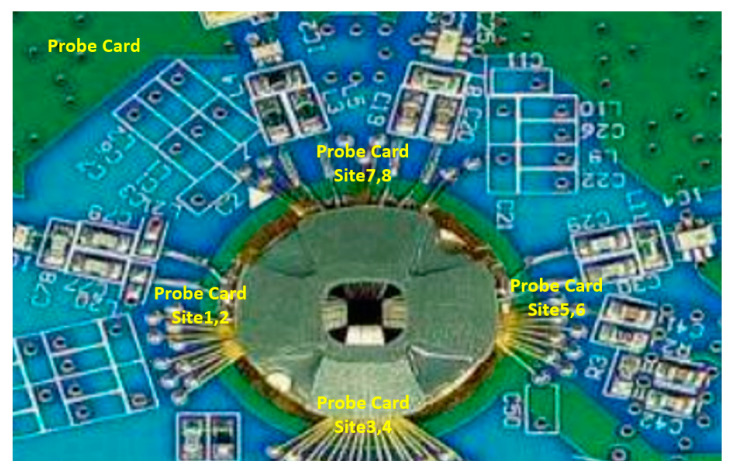
A wafer probing card.

During wafer probing, semiconductor devices are tested site by site, by measuring the variations in the electrical parameters at each site, such as voltage, current, and resistance, to ensure that they are functioning properly and meeting the required product specifications. If a defect occurs regarding the failure of electrical parameters at a specific site during mass production, then that wafer is held and binned. Figure 6 is an example of a wafer probing card.

Another type of failure commonly found in wafer probing is short failure, which occurs when two or more electrical connections on the device contact each other, creating an unintended connection. Wafer probing can detect such short circuits by measuring the electrical resistance. Conversely, open failure happens when an electrical connection is broken or disconnected, resulting in a lack of conductivity. Wafer probing detects such open circuits by measuring the electrical resistance. When open or short failures occur frequently during the wafer probing of mass-produced wafers, the affected wafer is held, and OCAP procedures are initiated.

Wafer probe yield measures the percentage of semiconductor devices that pass the probe test, indicating they are functioning correctly without any defects. The probe test yield is an important metric as it reflects the quality of the manufacturing process and the effectiveness of the testing methods. If the number of wafers placed in a bin for monitoring exceeds 1%, or if the resulting yield from wafer probing falls below 85%, then the probing lot must be put “on hold”. This action triggers the follow-up process, namely, the OCAP. Then, operators and engineers need to take proper actions to mitigate the situation.

Step 2. Operators’ and engineers’ actions taken.

If a problem of low yield is identified in any of the four issues mentioned above during the wafer probing test, then the operator immediately shuts down the wafer probing test equipment and reports the situation to the process engineer and maintenance team.

Step 3. Maintenance standard operating procedure (SOP).

In situations where the problem is due to the test hardware and tester setups, the connection status between the wafer probing head and the tester must be checked, along with the condition of the interface cable between the wafer probing head and the wafer probing card. Additionally, the pogo pin height and cleanliness should be inspected for any worn-out or broken pogo pins. Then, based on the SOP, equipment maintenance is performed. The SOP is described as follows:

(1) Is the failure due to the hardware?

- If yes, then identify test hardware to fix, then isolate and fix the problems, then perform a buy-off on a good unit with at least 10 touchdowns.
- If no, then move to the next step.

(2) Is the failure related to the wafer?

- If yes, then inform semiconductor fabrication plant (FAB) engineers.
- If no, then repair testers or probers, then perform a buy-off on a good unit with at least 10 touchdowns, then move to the next step.

(3) Is the bin failure corrected?

- If yes, then restart the process.
- If no, then good flow or reject scrap.

Once all issues have been addressed, a performance buy-off is conducted using the previous process flow. Before officially restarting the equipment and resuming production, at least 10 touchdowns (i.e., tests) must be conducted on quality products. The product engineer must review the probing data obtained. If there are no issues, the equipment can be reactivated, and production can continue.

## 3. The Proposed Novel OCAP

The OCAP is crucial for maintaining quality and efficiency in the semiconductor manufacturing processes. It provides a structured approach to identifying, analyzing, and correcting deviations, ensuring that the production meets stringent standards. By systematically addressing issues as they arise, the OCAP helps prevent minor problems from escalating into major defects, thereby safeguarding both the product quality and the production timeline.

Although the traditional OCAP has long assisted semiconductor manufacturing plants in maintaining production capacity, it is still intricate and demands significant resources, necessitating skilled personnel and strong systems to handle everything from data collection to the verification of corrective actions. These processes depend heavily on human judgment, which increases the likelihood of errors and biases. Additionally, they are labor-intensive and typically concentrate on immediate issues, neglecting wider systemic problems that could recur. Challenges in communication and a reliance on historical data can obstruct effective problem resolution and hinder adaptation to evolving conditions.

**Figure 7:**
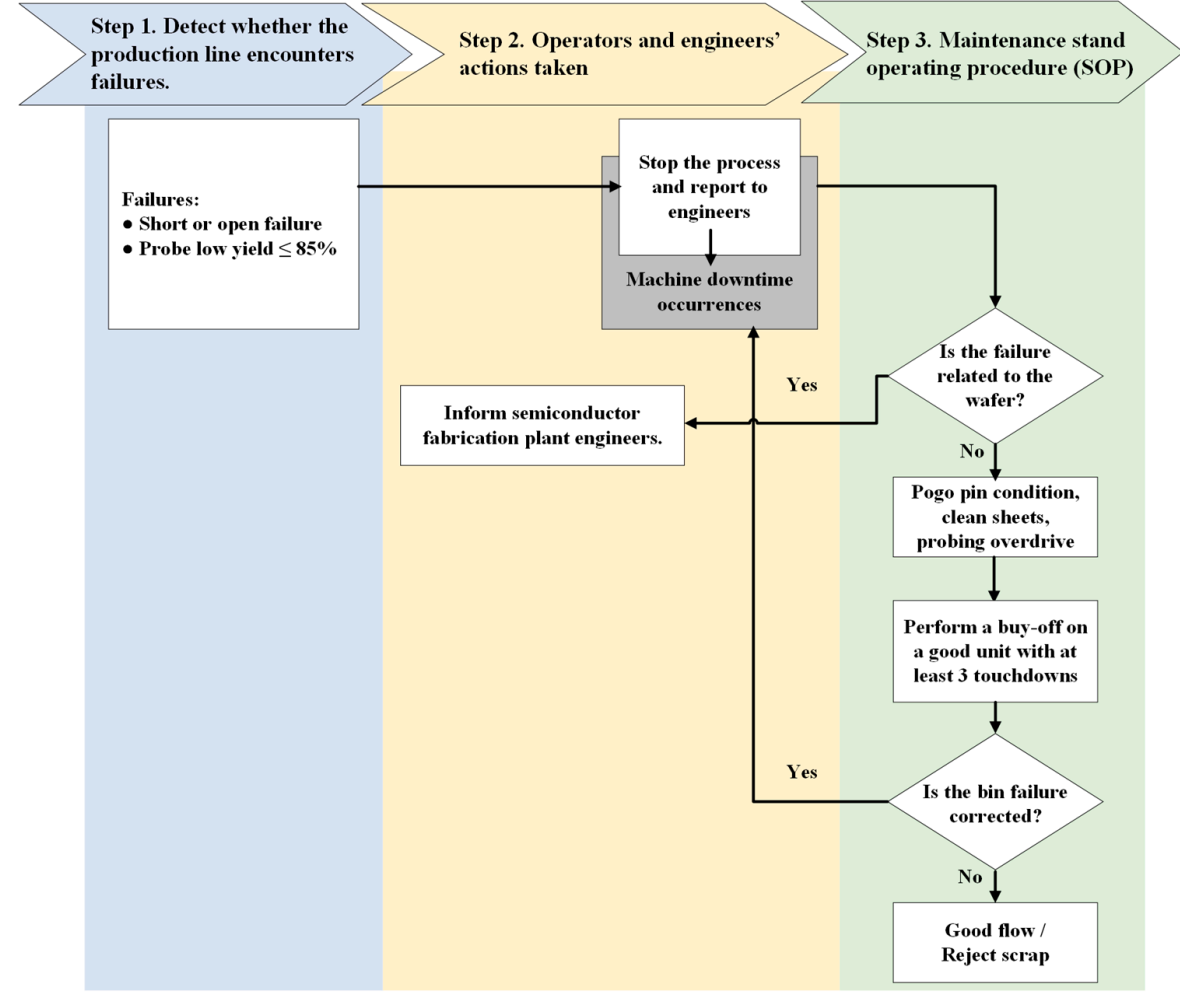
Flowchart of the proposed novel OCAP.

With the long-term operation of the traditional OCAP, semiconductor factories continue to collect relevant data at various work nodes. This paper, using data from an actual production line collected by a semiconductor factory in Taiwan, has developed a novel OCAP that simplifies traditional OCAP SOPs, reduces labor costs, and minimizes reliance on human judgment, thereby enhancing the execution efficiency of OCAP in the wafer probing process. The novel OCAP incorporates advanced data analytics to predict and preempt potential deviations before they escalate, ensuring higher accuracy and reliability. It integrates real-time monitoring systems and automated corrective action protocols, streamlining troubleshooting and ensuring consistent corrective measures. This proactive approach significantly shortens response times to anomalies, improves overall yield and throughput, and mitigates the risk of subjective biases, allowing skilled personnel to focus on strategic tasks. Additionally, the scalable framework of the novel OCAP can be adapted to various stages of semiconductor manufacturing and other high-precision industries, paving the way for future innovations in quality control and process optimization. The proposed novel OCAP can be divided into three steps (see Figure 7), and detailed descriptions of each step are as follows.

Step 1. Detecting whether the production line encounters failures.

The results shown in Table 1 are derived from one year of log data retrieved from the case company. The data summarize the frequency of occurrences that trigger the traditional OCAP, categorized by the root causes identified for lots with low probe yield. In Table 1, the *x*-axis lists the four main events that trigger the traditional OCAP, while the *y*-axis enumerates the key causes identified after implementing the OCAP. According to Table 1, the two events, “short or open failures” and “low probe yield”, account for over 60%, even over 70% of the triggers. Thus, when these events are compared with other causes that activate the traditional OCAP, “short or open failures” and “low probe yield” emerge as the primary issues prompting the OCAP, which in turn halts the wafer probing process.

The proposed OCAP identifies the main causes contributing to low probe test yield by analyzing the frequency of occurrences in the probe tester equipment repair history accumulated over one year. This analysis highlights the OCAP items with the highest frequency distribution among the major causes of low probe test yield. Unlike the traditional OCAP, which lists four failure items, the proposed OCAP focuses on the two failure items with the highest frequency: “short or open failures” and “low probe yield”. These items account for over 60% and even 70% of the triggers for the traditional OCAP, indicating their significant impact on overall yield and operational efficiency. By concentrating on these high-frequency issues, the proposed OCAP can more effectively allocate resources and streamline troubleshooting processes. Items not controlled under the proposed OCAP were found to have a negligible impact on overall yield and efficiency. In an industrial manufacturing context, controlling these less impactful items adds unnecessary complexity and resource expenditure. Therefore, the decision to exclude them aims to simplify the process and enhance its effectiveness. This targeted approach ensures that critical issues are addressed promptly and effectively, improving the overall efficiency and reliability of the wafer probing process. The influence of this streamlined control strategy is reflected in improved machine downtime and yield metrics, demonstrating the proposed OCAP’s effectiveness in maintaining high production standards. Thus, in this step, the production line faces a failure situation when reaching the following modified conditions:

- Short or open failures.
- Low probe yield ≤ 85%.

Step 2. Operators’ and engineers’ actions taken.

If a problem of low yield is identified in any of the two failures mentioned in step 1 during the wafer probing test, the operator immediately shuts down the wafer probing test equipment and reports the situation to the process engineer and maintenance team.

Step 3. Maintenance SOP.

Based on the SOP, perform equipment maintenance. The SOP is described as follows:

(1) Is the failure related to the wafer?

- If yes, inform FAB engineers.
- If no, check and adjust pogo pin conditions, cleaning sheets, probing overdrive, then, perform a buy-off on a good unit with at least 3 touchdowns.

(2) Is the bin failure corrected?

- If yes, restart the process.
- If no, good flow or reject scrap.

After analyzing the one-year log data of the probe tester on the number of touchdowns times performed before officially releasing the wafer prober (see Table 2), good units’ number of touchdowns is modified to three. If good units successfully pass with touchdown occurrences ranging from one to three during equipment buy-off, then it is also noted that the frequency of passing with touchdown occurrences from four to ten is notably high. It has been found that this accounts for 95% of the occurrences.

Once all issues have been addressed, the product engineer must review the probing data obtained. If there are no issues, then the equipment can be reactivated, and production can continue.

## 4. Results and Discussion

After applying the proposed OCAP to the production line of the case company, the data suggest that by refining and streamlining action items, the proposed OCAP process can greatly enhance machine availability and operational throughput. Table 3 provides a comparative analysis of a traditional and proposed OCAP process flow, focusing on different failure items in a testing scenario. The traditional process lists various action items associated with failure types and estimates the downtime for each tester. The proposed OCAP introduces efficiency improvements by either eliminating control steps or reducing the actions required. Significant reductions in downtime are noted across several categories. For example, in “perform buy-off (probe test condition check)”, the action reduces from “10 touchdowns” to “3 touchdowns”, reducing the downtime from 2.5 h to just 0.5 h. Similarly, in the case of “open or short failures”, the proposed flow reduces the downtime from 2 h to zero by setting a specific verification threshold. The overall effect of these process improvements is a substantial reduction in total downtime from 16 h in the traditional flow to 5.5 h in the proposed flow. This leads to an overall downtime reduction of 10.5 h, which represents a significant efficiency increase and potential cost savings in operational procedures.

To validate the effectiveness of the proposed OCAP, a production facility from the case company was selected for implementation. The facility’s downtime, OEE, and mass production wafer throughput were evaluated. A total of 13 lots, corresponding to 325 wafer probe data points, were analyzed. For comparison, 12 lots totaling approximately 300 wafers were randomly selected from existing facilities currently undergoing probe tests with the traditional OCAP. The downtime, OEE, and wafer throughput of this facility were benchmarked against those under the proposed OCAP conditions.

**Figure 8:**
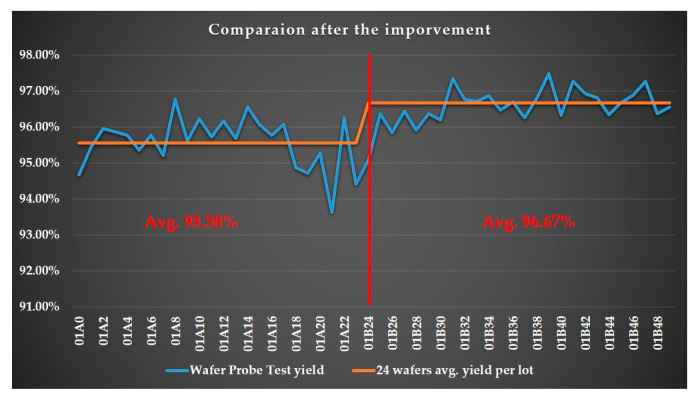
Comparing wafer probing yields: The traditional OCAP vs. the proposed OCAP.

Figure 8 presents the wafer probing yield for both traditional and proposed OCAPs at the selected facility. The *x*-axis represents the lot numbers in the study, while the *y*-axis indicates the wafer probing yield. A red reference line divides the graph, with yields from the traditional OCAP on the left and those from the proposed OCAP on the right. The analysis shows that the average test yield with the proposed OCAP is 1.1% higher than that of the traditional OCAP.

**Figure 9:**
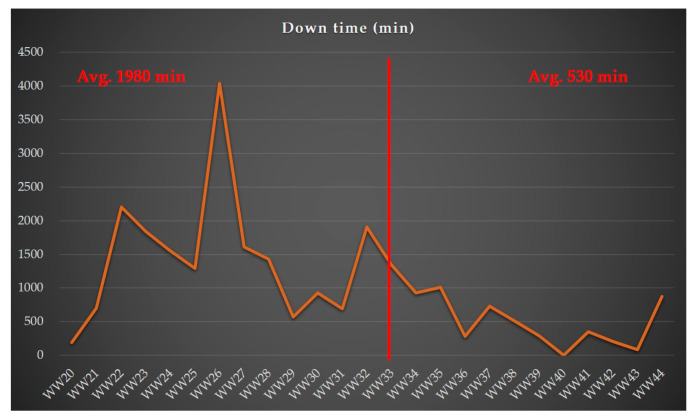
Comparing machine downtime: the traditional OCAP vs. the proposed OCAP.

Figure 9 depicts the weekly machine downtimes at the selected facilities under both the traditional OCAP and the proposed OCAP. The *x*-axis of the graph indicates the work weeks code and time duration, while the *y*-axis shows the machine downtimes. It is clear from Figure 9 that the average weekly machine downtime was reduced by 1450 min, equivalent to over 24 h, with the proposed OCAP compared to the traditional OCAP. This significant improvement in machine downtime under the proposed conditions contributes notably to enhancing output and OEE. Figure 9 illustrates the weekly machine downtimes at the selected facilities under both the traditional and proposed OCAPs. It is evident from Figure 9 that the average weekly machine downtime was reduced by 1450 min, equivalent to over 24 h, with the proposed OCAP compared to the traditional OCAP. This substantial reduction in machine downtime under the proposed conditions significantly enhances OEE.

Figure 8 and Figure 9 highlight the significant advantages of the proposed OCAP over the traditional OCAP. The comparisons underscore the proposed OCAP’s effectiveness in boosting yield and minimizing downtime, thereby improving the performance and reliability of semiconductor manufacturing processes.

**Figure 10:**
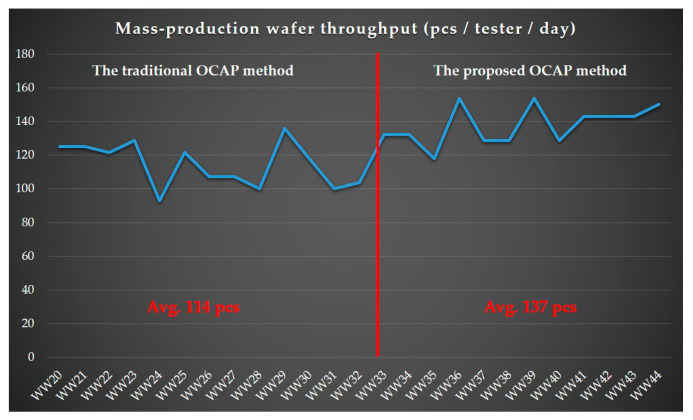
The best combination for effective throughput checks.

Additionally, Figure 10 presents a graph that compares the OEE and pack out between one of the facilities currently undergoing a probe test and lots managed under optimal OCAP process flow conditions. The *x*-axis of the graph indicates the work week code and time duration, while the *y*-axis shows the wafer probe test pack out. A red reference line divides the graph: the left side details the OEE and pack out for the facility under the traditional OCAP process flow (average 114 packs), and the right side shows the OEE and pack out for lots under the proposed OCAP conditions (average 137 packs). After analyzing over 300 instances of OEE and pack out, it is clear that the wafer probe test pack out for lots managed under the proposed OCAP process flow conditions exceeds those under the traditional OCAP process by more than 23 wafers per week. This significant boost in probe test pack out volumes under optimal conditions greatly enhances facility efficiency, fostering production growth and improving OEE.

Table 4 summarizes the effectiveness of two different OCAP processes by comparing three key performance metrics: average wafer probing yield, average machine downtime, and average throughput. The “level of best” is defined based on the performance improvements across these metrics under the proposed OCAP compared to the traditional OCAP, which analyzed as:

- Wafer probing yield: the average wafer probing yield under the proposed OCAP is 96.67%, compared to 95.58% for the traditional OCAP. This 1.1% increase is significant for the case company, producing about 15,000 chips per wafer. This increase translates to approximately 165 additional chips per wafer, adding significant value to the production process. The unit cost of the product for this effectiveness verification is USD 0.5, leading to a total added value of about USD 26.8K across 325 wafers.
- Machine downtime: The average machine downtime is reduced from 1980 min (traditional OCAP) to 580 min (proposed OCAP), equating to a reduction of 1400 min or about 24 h per week. This substantial decrease in downtime enhances operational efficiency.
- Throughput: The average throughput increases from 114 wafers per week (traditional OCAP) to 137 wafers per week (proposed OCAP), representing an improvement of about 23 wafers per week. This increase in production volume further highlights the effectiveness of the proposed OCAP.

## 5. Conclusions

The OCAP plays a crucial role in the wafer probing process within semiconductor manufacturing, serving as a fundamental mechanism for maintaining high standards of quality and efficiency. The traditional OCAP in semiconductor manufacturing faces several disadvantages that can hinder operational efficiency. Primarily, it is reactive rather than proactive, addressing issues only after they have occurred, which can lead to significant production delays and increased costs associated with diagnosing and rectifying problems. Moreover, traditional OCAP is often complex and resource-intensive, requiring significant human intervention, which increases the risk of errors and biases in problem-solving. Additionally, this approach can be time-consuming, as it involves multiple steps from data collection to the implementation of corrective actions, slowing down the overall production process. Furthermore, traditional OCAP generally focuses on immediate problems without addressing underlying systemic issues, leading to the potential recurrence of the same problems. To address these issues, this paper has successfully developed and evaluated a novel OCAP for semiconductor manufacturing, focusing on the wafer probing process. The implementation of this novel OCAP has demonstrated quantifiable improvements: it has reduced machine downtime by over 24 h per week and increased wafer production volume by approximately 23 wafers per week, while also enhancing the probe test yield by an average of 1.1%. These results not only signify a marked enhancement in operational efficiency but also underscore the substantial contribution of the novel OCAP to increasing production capacity and improving OEE. However, this paper is not devoid of limitations. The data and outcomes are based on implementation within a single production facility, which might not fully represent other environments with different operational dynamics or technological setups. Therefore, the findings might exhibit limited generalizability across different semiconductor manufacturing contexts or to other industries with similar processes.

## Figures and Tables

**Table 1:** Occurrence frequency for the traditional OCAP process flow control items vs. low probe test yield root causes.

Root Causes Found	Events to Trigger the Traditional OCAP				
	Line Pattern	Site Issue	Short or Open Failures	Low Probe Yield	Total
Probe test setup or probe test conditions issue.	13.7%	15.7%	37.2%	33.4%	100%
			70.6%		
Probe card for any defects, wear and tear, or contamination that could affect the test results.	7.9%	21.6%	30.2%	40.3%	100%
			70.5%		
Device under test issues or FAB process defect.	20.8%	6.9%	36.6%	35.7%	100%
			72.3%		
Probe test equipment is properly calibrated and functioning correctly.	10.6%	15.2%	28.1%	46.1%	100%
			74.2%		
The test process itself may be poorly designed or not optimized for the specific device being tested.	20.1%	19.4%	28.2%	32.3%	100%
			60.5%		

**Table 2:** Good units’ touchdown time vs. occurrence frequency during probe tester buy-off.

Item	Good Units Touchdown Time				Occurrence Frequency
	1 Time	2 Times	3 Times	4–10 Times	
Good units’ touchdown result	Pass	Pass	Pass	Fail	<5%
	Pass	Pass	Pass	Pass	>95%

**Table 3:** Comparing the traditional OCAP process flow with the proposed OCAP process flow.

	The Traditional OCAP Process Flow		The Proposed OCAP Process Flow		
Failure Items	Action Items	Estimated Tester Down	Action Items	Estimated Tester Down	Summary for Reducing Machine Downtime
Line pattern	Verify line pattern	3 h	No control	0	3 h
Site issue	Verify site issue	3 h	No control	0	3 h
Open or short failures	Verify open, short failure	2 h	Verify open, short ≥1%	2 h	0 h
Low probe yield	Verify low-yield issue	3 h	Verify low-yield issue	3 h	0 h
Perform buy-off (probe test condition check)	10 touchdowns	2.5 h	3 touchdowns	0.5 h	2 h
Check for H/W problem	Verify H/W problem	2.5 h	No control	0	2.5 h
Total		16 h (960 min)		5.5 h (330 min)	10.5 h (630 min)

**Table 4:** The best summary of effectiveness combinations.

	Average Wafer Probing Yield	Average Machine Downtime	Average Throughput
The traditional OCAP	95.58%	1980 min	114 wafers
The proposed OCAP	96.67%	580 min	137 wafers

## Data Availability

Data available on request due to restrictions.
